# Supplementary material for: Are carbon emissions trading and green financial instruments synergistic? -Comprehensive quantitative research based on content analysis
Source: PLoS One. 2024 Mar 7;19(3):e0298601. doi: 10.1371/journal.pone.0298601 (PMC10919601; doi:10.1371/journal.pone.0298601)
Supplement: S1 Data — (ZIP) [file pone.0298601.s001.zip › Data/地级市Z_F工作报告.zip!/╣π╢1⁄2/╘╞╕í╩╨/└┤╫╘╣1⁄2╓┌║┼í╛┬φ┐╦╩2╛▌═°í┐_2011.docx]

各位代表：

我代表市人民政府向大会作政府工作报告，请予审议，并请政协各位委员和其他列席人员提出意见。

“十一五”时期工作回顾

2006年以来,市政府在省委、省政府和市委的正确领导下，在市人大及其常委会和市政协的监督、支持下，高举邓小平理论和“三个代表”重要思想伟大旗帜，围绕建设全省农村改革发展试验区、循环经济和人居环境建设示范市、广东富庶文明大西关的战略定位，坚持以科学发展观统领经济社会发展全局，积极探索科学发展新模式，坚决贯彻执行中央“保增长、保民生、保稳定”和省“三促进一保持”、“双转移”等重大决策部署，大力推进“两区四城一纽带”建设，圆满完成“十一五”经济社会发展主要预期目标任务。我们兑现了近几年《政府工作报告》中提出的加快发展承诺，不仅在解决长期制约云浮发展的交通基础设施、工业发展载体等瓶颈问题上取得重大突破，在人居环境建设、农村改革发展、“三网融合”、农村金融创新、扶贫开发、新型农村合作医疗等方面也一直走在全省乃至全国的前列，不断创造新鲜经验，我市的发展条件已经发生了根本性的变化，已形成了前所未有、生机勃勃、加快发展的良好态势，我们将在新的发展起点上描绘科学发展、跨越发展、和谐发展的美好蓝图。

2010年，全市实现生产总值394.27亿元，比上年(下同)增长13.8%，比2005年增长83.6%；万元生产总值能耗1.27吨标准煤，比上年降低1.52 %；规模以上工业增加值130.83亿元，增长31.6%，比2005年增长196.1%；农业总产值164.8亿元，增长5.9%，比2005年增长32.9%；全社会固定资产投资总额312.66亿元，增长30.2%，比2005年增长191.4 %；外贸出口总额8.13亿美元，增长32.1%，比2005年增长88.6 %；实际利用外资7929万美元，增长11.5%，比2005年增长108.7%；地方财政一般预算收入23.54亿元，增长25.4%，比2005年增长152.3%；城镇居民人均可支配收入14613元，增长10.6%，比2005年增长56.5%；农民人均年纯收入6744元,增长16.9%，比2005年增长59.5%。2010年全市60项重点工程（项目）新投入144.86亿元，基本完成年度投资计划。

——基础设施建设取得重大突破。全力推进以“五三二一”工程为重点的交通基础设施建设。五年来，全市共投入交通基础设施建设资金172.69亿元，建成公路通车总里程7110.9公里，分别是“十五”时期的4.7倍和1.6倍。2007年提前两年实现村村通硬底化道路。广梧高速公路（河口至平台段）、河杨公路、云浮新港和城市候机楼等先后建成投入使用，一批县通镇公路实现了硬底化；国道324线云浮段、云六公路、省道368线六都至平台段和省道352线南江口至大湾段完成大修，省道280线罗定段、省道369线罗定段和省道276线新兴县城至集成段完成改造。江罗高速、罗阳高速前期工作进展顺利，争取今年动工建设；汕湛高速云浮段已完成工程可行性报告编制；南广高铁正在加紧建设，2012年底建成通车；罗岑铁路累计完成投资4.8亿元。都骑通用码头初步设计已获省交通运输厅批复。温氏码头正在开展项目深度论证工作。沿江公路和云浮新港物流产业基地等重点工程项目较快推进。

——工业集聚发展取得重大突破。落实《云浮市特色工业发展规划（2009-2015年）》，组织实施千亿工业大会战和企业成长工程，特色工业加快集聚发展的态势已经形成。获批了石材、不锈钢制品和电池等3个省级产业集群升级示范区。国家石材产品质量监督检验中心和五金制品、石材省站基本建成，省级五金制品和石材专业标准化技术委员会落户我市。石材产业年产值达78亿元，年缴税1.17亿元，建成东骏保税仓、石材博览中心和百里通、新富云、海岸石业、好世界等一批较大规模的石材企业，投资达50亿元的中国·云浮国际石材产业城项目已签约启动。淘汰落后水泥产能593万吨，建成先进产能800多万吨，天山水泥、中材亨达水泥一线、二线和中材（郁南）水泥项目先后建成投产，中材（罗定）水泥、华润（罗定）水泥、青洲水泥、中材亨达水泥粉磨等项目先后动工。不锈钢制品产业年产值达到63.2亿元，比2005年增长146.9%，先丰东成分厂、三A餐具城、国联餐具、裕隆公司、德纳五金制品等一批项目建成投产。与省共建的先进制造业（硫化工）产业基地建设加快，惠沄化工基地、惠沄钛白扩建、联发硫酸、金泰硫酸、银利化工、创东硫酸等一批项目建成投产。电力产业装机容量达到180万千瓦，投资27.9亿元的云浮发电厂C厂已建成投产，总投资10.7亿元的云浮风电项目（罗定亚婆髻山和新兴天露山风电场）获省发改委核准，今年上半年可望动工，总投资50亿元的华润西江发电厂首期项目已上报国家能源局；云浮核电站项目正抓紧申报进入国家和省的核电发展规划。同时，在“十一五”期间，全市共新增规模以上企业324家。成功举办了第三届至第七届中国（云浮）国际石材科技展览会，云城区先后获得“广东省民族民间（石雕）艺术之乡” 、“中国人造石之都”、“中国民间文化（石雕）艺术之乡” 称号。打造驰名商标2个，实现了驰名商标零的突破。温氏集团被认定为国家级企业技术中心，云安县被授予国家可持续发展实验区和省循环经济试点县，云硫集团被授予国家级循环经济发展试点单位。

——农村改革发展取得重大突破。大力建设全省农村改革发展试验区，全面推进农村“六项改革”，实施农村发展“十大工程”。 在云安开展农村综合改革、在郁南开展农村金融改革、在新兴和云城开展农村居民养老保险试点、在罗定开展户籍制度改革试点工作，并全力打造国家级畜牧业产业化示范中心。云安县成为中国改革（2010年）年度十大县市。省主要领导对我市在农村改革方面的创新突破给予了充分肯定。农村饮水安全、水库除险加固、城乡防灾减灾、地质灾害防治、生态防护林、现代农业园区、沼气池、医疗卫生等工作取得新成效。建成无公害农产品、绿色食品和有机食品生产基地49个，面积达35.1万亩，郁南县10万亩沙糖桔、罗定市10万亩水稻生产基地被列为全国绿色食品原料标准化基地；全市获得无公害农产品认证39个、绿色食品认证21个、有机食品认证8个，农业类广东省名牌产品7个，健康农业示范区20个。2010年全市农业产业化经营组织达到285个，比2005年增加150个；实现年销售收入259.2亿元，比2005年增长150%；带动合作农户户均增收超过7900元，比2005年增长88%。温氏集团成功拓展生物制药等产业，并入选2010年广东省自主创新百强企业，2010年实现销售收入219.4亿元，是2005年的3.5倍。粮食工作连年完成省下达的任务。利用低效园地、山坡地开发补充耕地5.11万亩。解决63.2万人农村饮水安全问题，完成190宗农村机电排灌议案工程，基本完成13宗城乡防灾减灾和158宗病险水库加固工程建设。

——“双转移”工作取得重大突破。制订优惠政策，落实招商引资责任制和激励机制，全面推进招商引资。五年来，共签约项目976个，合同投资总额968亿元。其中2010年,全市签约项目333个，合同投资总额282.2亿元，是“十五”期间总引资额（140亿元）的2倍多。实施共建共享机制，重点打造的佛山（云浮）产业转移工业园、佛山顺德（云浮新兴新成）产业转移工业园、云浮循环经济工业园和罗定双东环保工业园（含郁南大湾分园）等“四大园区”已逐渐成为全市经济的新增长点。佛山（云浮）产业转移工业园成功评为省第三批示范性产业转移工业园，竞获省竞争性扶持资金5亿元。佛山顺德（云浮新兴新成）产业转移工业园竞获了专业性产业转移工业园扶持资金1亿元。2010年“四大园区”基础设施建设新投入26.59亿元，引入项目56个，计划投资总额160.98亿元。成功申报设立省级高新技术产业开发区。推进农村劳动力转移，抓住市技工学校被评为国家重点技工学校的契机，不断扩大市技工学校实训基地及其他中职培训机构规模，五年内共新增转移农村劳动力22.12万人，组织技能培训13.26万人，培训后转移就业率达90%。我市“技能下乡”模式得到省委、省政府的充分肯定。2010年罗定市被评为“全国农村劳动力转移就业工作示范县”。

——宜居城乡建设水平显著提升。全力推进“美好环境与和谐社会共同缔造”，增强市民对云浮故乡的认同感和归属感。围绕做大做优中心城区，努力打造“健康、生态、幸福”的宜居城乡，以17个共建项目为抓手，推进云城、云安同城化，“一江三组团”城市格局初步显现，城市面貌焕然一新，南山森林公园一带成为市民喜爱的好去处。已建成开通164公里绿道。完成了8个宜居城镇、63个宜居村庄、16个宜居社区的试点创建工作，建成生态文明村2819条，受惠群众约72.6万人。“三旧改造”稳步推进，完成了城区主要道路升级和部分供水管网改造，并成功创建省级卫生城市。罗定、新兴、郁南、云安等中心城镇建设步伐加快，大大提升了各地的人居环境质量和城市品位。成功创建省林业生态市，五个县（市、区）均已成为省林业生态县，郁南县、罗定市被授予“全国绿化模范县”。2010年，全市森林覆盖率66.5%，比2005年提高了5.4个百分点。率先建立“三规合一”机制。国家有关机构和专家学者对我市人居环境建设进行了研讨，给予了充分肯定，并发表了《美好环境与和谐社会共同缔造——云浮共识》。

——现代服务业发展水平显著提升。率先推进“三网融合”，已实现8万用户三网融合双向互动数字电视整体转换，并以三网融合为平台，积极发展远程医疗、远程教育、物联网以及相关产品研发、生产。2010年，我市被批准为中国电子商务三网融合创新示范城市、省三网融合双向互动数字电视应用试点市和省远程医疗工作试点市，我市三网融合应用示范产业基地列入广东省首批战略性新兴产业基地。完善旅游景区基础设施和服务设施建设，积极开拓禅宗文化生态观光、休闲度假、乡村民俗等特色旅游。“十一五”期间全市接待游客总人数和旅游总收入分别是“十五”时期的2.63倍和2.59倍。六祖故里旅游度假区和金水台温泉度假区成为国家4A级旅游景区，新兴县被评为广东省旅游强县。实施“万村千乡市场工程”，组织家电、建材等下乡活动，激活了农村消费。2010年，全市社会消费品零售总额136.97亿元，增长16.5%，比2005年增长129.4 %。

——财税金融支撑能力显著提升。2010年，全市国税收入17.1亿元，比2005年增长72%；地税收入18.2亿元,比2005年增长108%。地方财政一般预算收入23.54亿元，比2005年增长152.3%。金融机构存贷款持续增长，本外币存款余额486.93亿元，比2005年增长123.98 %。贷款余额278.34亿元，比2005年增长186.65%。存贷比达到57.16%，比2005年提高了12.5个百分点。成立了广东省农村信用联合社云浮办事处和邮政储蓄银行云浮分行。政银企合作取得重大成果，我市与金融机构签订的授信总额共566.8亿元的融资协议，已落实贷款219.04亿元；各金融机构与“五大”百亿产业集群对接融资项目2586个，已发放贷款87.39亿元。广东大华农动物保健品股份有限公司已批准上市，广东凌丰集团股份有限公司已完成上市备案工作。同时，我市成为全省首个创建信用体系试点市。

——社会民生保障水平显著提升。五年来，我们致力推进城乡居民基本医疗保障工作，成功创建广东省农村中医工作先进市，深入推进卫生体制改革，全市78%的镇卫生院和社区卫生服务机构实施国家基本药物制度，建设3.9万户农村卫生厕、217条省级卫生村，完成600间农村卫生站规范化建设以及20间薄弱卫生院改造任务。“双档型”农村合作医疗制度获得“中国十大政府创新典型”奖。启动远程医疗项目，有效解决群众看病难、看病贵问题。全市城乡低保救助、五保供养、优抚对象抚恤补助自然增长机制进一步健全。成立了市县两级慈善会，筹集捐款8800多万元。职工基本养老保险、被征地农民养老保险已全覆盖,六大险种实现市级统筹，金保工程网络完成省到中心镇的四级联网，成为全国金保工程建设示范城市，向着人人享有基本养老保险目标迈出一大步。全市投资6000多万元建设各类保障性住房5万多平方米，解决了1600多户城镇低收入家庭的住房困难问题。扶贫开发“规划到户、责任到人”工作全面铺开，全市87个贫困村和3.2万户贫困户全部得到切实帮扶，42.8%贫困户2010年实现年人均纯收入2500元以上的脱贫目标。“十一五”期间全市农村贫困人口减少近10万人。我市金融扶贫、智力扶贫、产业扶贫和社会保障扶贫模式得到省委、省政府的充分肯定，扶贫工作在2010年省组织的综合考评中获得“优”的等次。2010年获中国产学研合作促进奖。5年来获批省级以上科技项目365项，其中省部产学研合作项目79项。获国家科技奖2项，省科技奖19项。设立高校研究院2个，创建省级工程技术研究开发中心8家、农业科技创新中心4家、农业科技园1个、专业镇20个。落实各级各类教育助学政策，受惠学生188万人次；完成教师“代转公”工作，落实教师工资福利待遇“两相当”；2010年高中阶段毛入学率82.27%，比2005年提高48.82个百分点。高考上本科人数达到5598人，比2005年增加1769人；市特殊教育学校建成；市教育园区建设全面启动。我市获“全国电化教育先进单位”和“广东省农村教育信息化示范市”称号。积极建设文化强市，大力打造禅宗六祖文化、南江文化和石艺文化品牌，“广东禅文化创意产业园”和“广东石文化创意产业园”获省批准。“六祖惠能”被评为“岭南文化十大文化名片”之一，“禾楼舞”列入国家级非物质文化遗产保护名录，罗定市、新兴县被授予“全国文化先进县”。建设完善城市规划演示馆、市博物馆、图书馆和档案馆等展馆。农村电影放映、广播电视村村通、农家书屋等惠民工程不断落实。参加十三届省运会获26枚金牌，名列全省12位。人口与计划生育工作进入全省先进行列，2008、2009年、2010年连续三年被省政府授予“广东省人口与计划生育先进单位”，新兴县被授予“全国计划生育优质服务先进单位”称号。环境质量继续改善，主要污染物排放量控制在省下达的考核指标以内。珠江整治考核名列全省前茅，2007年获全省第一。市区空气优良率达99%。提前实现县县建成污水处理厂目标。大力实施食品药品放心工程，市场秩序进一步规范。连续多年完成了省下达的安全生产各项任务，社会治安综合治理“平安和谐”工程深入推进，市、县、镇三级综治维稳工作平台得到进一步巩固完善，镇街综治信访维稳中心建设走在全省前列，齐抓共管的治安综治工作机制进一步磨合到位，圆满完成了奥运会、亚运会、亚残运会召开期间的安保和维稳任务，社会治安秩序稳定。

——政府执行力显著提升。认真落实市人大及其常委会各项决议、决定并向其报告工作，广泛听取人大代表、政协委员和各民主党派、工商联、无党派人士的意见建议。五年来共办理市人大建议511件、市政协提案798件。创新项目建设抓落实和激励机制，市领导班子成员挂点重点工程(项目),明确责任,推动落实。完成了市级政府机构改革。完成市本级政府第三、第四轮行政审批事项清理和调整。认真落实廉政建设制度，对党政班子和领导干部开展落实科学发展观实绩考核，进一步提升了各级各部门推进科学发展跨越发展的自觉性和执行力。

此外，物价、统计、审计、人事人才、编制、知识产权、民族宗教、档案、外事侨务、港澳事务、台务、人民防空、地方志、消防、保密、气象、侨联、文联、社科联、妇儿、科协、工商联、红十字会、贸促会、新闻出版以及驻市外机构等工作也取得新成绩。

过去五年，我们沉着应对国际金融危机和自然灾害等严峻挑战，积累了在复杂困难的环境中抓准机遇、化危为机，推动经济社会科学发展跨越发展的经验：一是必须坚持以科学发展观为引领，敢于率先探索和实践符合云浮实际的科学发展模式和路径，敢于自加压力，迎难而上，在工作中创造新业绩，实现新突破；二是必须坚持以“双转移”和项目建设为抓手，加快做大总量，夯实加快发展的基础；三是必须坚持以体制机制创新为突破口,增强加快发展的动力；四是必须坚持以惠及民生为出发点, 凝聚美好环境与和谐社会共同缔造的合力；五是必须坚持以转变作风为重点,推动学习创新，创建学习型机关，切实提高干部队伍的执行力。

各位代表！过去五年政府工作取得的成绩，来之不易。这是在省委、省政府和市委正确领导下，全市上下抢抓机遇、开拓进取、共克时艰、奋力拼搏的结果。在此，我代表市人民政府，向全市人民、驻云浮部队官兵和关心支持云浮发展的港澳台同胞、海外侨胞及国际友人表示衷心的感谢并致以崇高的敬意！

在总结成绩的同时，我们也清醒地看到，我市经济社会发展中仍存在不少困难和问题：发展不充分，经济总量小；基础设施建设仍然不能满足经济社会发展的需要；基本公共服务不均衡，改善民生任务繁重；政府服务效能有待提高。对此，我们一定高度重视，采取有效措施，在今后的工作中努力加以解决。

“十二五”时期的指导思想、发展目标和主要任务

“十二五”时期，和平、发展、合作仍是时代潮流，国内经济形势总体向好。我省将围绕“加快转型升级，建设幸福广东”这个核心，深入实施《关于促进粤北山区跨越发展的指导意见》，继续加大统筹区域协调发展的力度，推动全省经济社会持续较快发展。今后五年，是我市乘势而上、推动经济社会跨越发展的重要战略机遇期，我们必须紧紧抓住发展机遇，以干大事、创大业的气魄，振奋精神，扎实工作，全力推动经济社会又好又快发展。

“十二五”时期的指导思想：高举中国特色社会主义伟大旗帜，以邓小平理论和“三个代表”重要思想为指导，深入贯彻落实科学发展观，以科学发展为主题，以加快转变经济发展方式为主线，围绕建设“幸福云浮”这个核心，深入实施“集聚优势、特色主导、中心崛起、城乡统筹、绿色生态”战略，全面推进云浮市美好环境与和谐社会共同缔造行动，大力实施“十大幸福计划”和“十大发展工程”，加快建设全省农村改革发展试验区、循环经济和人居环境建设示范市、广东富庶文明大西关，开创云浮科学发展、跨越发展、和谐发展新局面。

“十二五”时期的发展目标：地区生产总值年均增长15%左右，地方财政一般预算收入年均增长20%以上，全社会固定资产投资年均增长28%以上，可持续发展能力显著提升，现代化生态滨江新城架构初步形成，社会事业全面发展，人民生活幸福安康。

“十二五”时期的主要任务：围绕建设“幸福云浮”这一核心，大力实施集聚优势、特色主导、中心崛起、城乡统筹、绿色生态“五大战略”，着力推进收入倍增、教育提升、文化惠民、医疗健康、社保扩面、安居保障、扶贫开发、基层善治、宜居城乡、平安云浮“十大幸福计划”和基础设施拓展、传统产业升级、新兴产业培植、现代农业提升、现代物流整合、旅游品牌打造、重点园区共建、信用云浮成长、创业人才集聚、体制机制创新“十大发展工程”建设，形成中等城市规模的中心城区、规模化现代产业园区和生态宜居城乡，推动经济社会在“十二五”期间实现新跨越。

2011年工作安排

今年是“十二五”开局之年。政府工作的总体要求是：全面贯彻党的十七届五中全会、中央经济工作会议、省委十届八次全会和市委四届九次全会精神，深入贯彻落实科学发展观，以加快转型升级、建设“幸福云浮”为核心，以加快转变经济发展方式为主线，以实施《云浮市改革发展规划纲要（2009-2020年）》和《美好环境与和谐社会共同缔造行动纲要》为抓手，全力推进“十大幸福计划”和“十大发展工程”，为“十二五”发展开好局、起好步。

2011年全市经济社会发展的预期目标是：生产总值增长15%；地方财政一般预算收入增长25%；社会消费品零售总额增长16%；全社会固定资产投资增长30%；城镇居民人均可支配收入、农民人均纯收入分别增长12%和11.84%；万元生产总值综合能耗、主要污染物排放量控制在省下达的目标以内；城镇登记失业率控制在3.5%以内；居民消费价格总水平上涨幅度控制在4%以内。

实现上述目标，必须抓好以下六方面工作：

一、全力扩大内需，促经济提速增长

抓住扩内需机遇，实施基础设施拓展工程，加大生产性投资和基础设施建设投入，进一步稳定物价和扩大消费需求，保持全社会固定资产投资一个时期的高速增长，对促进云浮跨越发展有着十分重要的战略意义。今年安排市重点工程（项目）100项，总投资约797.1亿元，其中年内完成投资209.88亿元。

加快交通基础设施建设。今年计划总投资70.2亿元。在重点加快“七三三三一”工程（即七条高速：广梧、云岑、阳罗、江罗、怀罗、罗信和汕湛清远清新至云浮新兴段；三条铁路:南广高铁、罗岑铁路、肇云轻轨；三个码头:云浮新港码头、都骑通用码头、温氏码头；三大物流园:新港物流园、都杨物流园、温氏物流园；一段西江黄金水道）建设的同时，重视整合铁路、公路、港口码头、物流园区和客运站场建设，推动各类运输资源共享、无缝对接，构建云浮市综合交通运输体系，提高交通设施的经济效益和社会效益。云岑高速公路完成投资17.1亿元，双凤至罗定段争取年内基本建成。沿江公路（六都至都杨段）完成投资1.1亿元，争取年内基本建成。完成西江航道（云浮段）整治工程；南广高铁（云浮段）完成投资24.5亿元，罗岑铁路完成投资6亿元；阳罗高速公路、江罗高速公路和新城快线确保年内动工；加快国道324线（安塘至河口）改线工作；加快省道113线、276线、352线的升级改造。全力推进后备项目立项攻关。加快怀茂高速（云浮段）和肇云轻轨、都杨西江特大桥项目的前期工作。

加大工业投资。扩大对新型工业项目的投资，争取工业投资不低于全社会固定资产投资增幅。列入今年100项重点工程（项目）的50项生产性工业项目确保完成投资86亿元以上。中材（罗定）水泥、华润水泥一期、贝铝首期、中材亨达400万吨粉磨站、广云铝板首期、贞英木业、新顺景陶瓷、汇得力陶瓷首期、绿量电池、东润石材、立伟达石料制品等项目要建成投产，宏佳铝业、发恩德高村铅锌矿首期项目争取基本建成，青洲水泥首期、罗定风电、新兴风电等项目加快建设，温氏嘉吉、新合铝业、环科电解锰和硫酸、云硫集团球团矿、中国·云浮国际石材产业城、中交二航水泥预制件等项目确保动工。继续促签约项目尽快落地动工，做好重大项目储备。加快推进华润西江发电厂一期项目，做好云浮核电、云浮电厂D厂、粤泷电厂二期等项目前期工作。

加强农村基础设施建设。落实2011年中央一号文件精神，加快建设民生水利工程。扎实开展全国水利普查工作。加强灌区水利工程及配套设施建设，着力恢复和改善农田灌溉系统，保障农业灌溉用水。加快城乡水利防灾减灾工程和病险水库除险加固工程建设进度。积极推进有防洪任务的中小河流整治，逐步推进各县（市、区）小流域综合治理工程、山洪地质灾害防治工程和大中型病险水闸除险加固工程建设。加强水资源优化配置和节约保护。加快完善农村公路和相关配套设施，改善群众出行条件。

加快推进城镇化。实施宜居城乡计划，深入推进 “健康、生态、幸福”宜居城乡建设，加快提升中心城区、县城、中心镇的交通、通信、供电、供排水、垃圾处理等基本公共服务保障水平。继续推进云城云安同城化，加大对城区交通的规范和整治，加快创建国家卫生城市步伐，提升城市管理水平。建设中心城区粪便处理系统，确保处理率达90%以上。完善优化公交线路和慢行系统，鼓励市民践行低碳生活。继续加强城镇环境整治，大力推进“三旧”改造及城市综合体项目建设，促进房地产健康发展。全面实施绿色生态工程，年内创建宜居城镇8个、宜居村庄70条、宜居社区16个。实施安居保障计划，加快保障性住房建设。

积极扩大消费。大力开拓绿色消费、健康消费、文化消费、信用消费等新兴消费热点，抓好“万村千乡”市场工程。落实汽车、家电、建材下乡等促进农村消费的措施。积极参加“广货全国行”活动，加快引进家乐福等品牌流通企业，拓展内销平台。实施旅游品牌打造工程，积极发展禅宗文化游、温泉休闲游、绿道体验游、乡村观光游、农家乐等旅游项目。探索利用农村信用平台融资加快农民自有住房升级改造的途径，拉动农村住房消费，不断拓展新的消费领域。

着力保持物价稳定。着力加强“米袋子”、“菜篮子”工程，保障主要农产品、基本生活必需品、重要生产资料的有效供给，切实控制商品价格过快上涨，落实稳定房价的各项政策，稳定消费价格总水平，保障群众的基本生活。加强市场监管，严厉打击囤货居奇、哄抬价格行为和制假售假、走私贩私等活动。

二、全力推进工业会战，加快构建现代产业体系

把做大做强做优工业作为现阶段加快转型升级、加快推进现代产业体系建设的首要任务来抓。全力推进千亿工业大会战，继续实施企业成长工程，加快打造“五大”百亿产业集群，努力推动工业率先实现跨越发展。

大力改造提升优势传统产业。加快实施市政府与省经信委签订的共同推进优势传统产业转型升级行动方案，实施传统产业升级工程。年内新增规模以上工业企业100家，限额以上商贸服务业企业100家。通过产业规划、环保措施等经济手段加快中心城区石材企业搬迁以及原有散乱石材企业的整治，打造好石材电子商务交易中心，办好第八届中国（云浮）国际石材科技展览会。以循环经济发展理念提升做强水泥和硫化工产业。加快水泥粉磨站项目建设，实现水泥熟料生产能力和粉磨能力相配套，明年基本实现水泥熟料市内粉磨。继续做好石灰石矿山的勘探和开采调配，促进水泥产业的可持续发展。加快推进省市共建广东省（云浮）硫化工先进制造业基地建设，继续拉长硫酸下游产业链条。引导扶持不锈钢制品产业优化产品设计，提升产品科技和文化含量，加快创高端品牌、国际品牌步伐。争取凌丰集团上市。做好电力储备项目的前期工作。继续抓好电池和新型建材产业的在建项目，加快佛山（云浮）建材产业转移转型升级示范区一期工程建设。实施现代物流整合工程，充分发挥西江黄金水道、铁路、高速铁路和公路、高速公路的物流效能，加快发展物流产业。

加快培育战略性新兴产业。实施新兴产业培育工程。大力培育“三网融合”应用产业和健康产业。以加快建设云浮“三网融合”应用示范产业基地为抓手，着力推进温氏物联网、远程教育、远程医疗、容灾备份数据中心等重点项目建设，积极引进相关的研发、生产企业落户我市并加快集聚发展。加快发展三网融合数据交换、数字家庭电子产品制造等三网融合应用产业，推动迅速形成三网融合新业态。以大华农公司生物制药和医疗康复器械制造项目为起步，加快形成健康产业的业态和规模。加快与省经信委签订“省市共建战略性新兴产业协议”。

加快“四大园区”发展。推进佛山（云浮）产业转移工业园、佛山顺德（云浮新兴新成）产业转移工业园、云浮循环经济工业园和罗定双东环保工业园（含郁南大湾分园）建设，突出抓好“三完善一提升”，即完善园区基础设施、完善园区征地及报批手续、完善公共配套服务设施，提升园区产业集聚发展能力。基本建好园区道路、供电、供水、通信、污水处理、生活服务小区等设施，并开通公交线路。按照各园区产业发展定位，设定入园项目的投资强度、投资规模和投资回报条件，年内新动工和建成项目的投资额实现30%以上增速。佛山（云浮）产业转移工业园和佛山顺德（云浮新兴新成）产业转移工业园要力争成为省重点扶持产业转移园。

实现招商引资新突破。加大招商引资力度，完善和落实招商引资工作责任考核和激励机制，提高招商引资水平。加强对国企、世界五百强、大型民企和各行业协会、商会的招商推介，积极参加省组织的招商活动，办好面向“三网融合”相关应用产业和医药、医疗康复器械制造产业的专场招商活动。各地围绕各园区的主导产业安排相应的专场招商活动，进一步提高引资成功率。年内佛山（云浮）产业转移工业园管委会和各县（市、区）引资额争取在去年的基础上增长30%以上，完善和兑现招商引资奖励政策。

加强自主创新能力建设。积极推进产学研合作，借助企业科技特派员、创新联盟、科研院校的力量，发挥好云浮高新区、民营科技园、专业镇、技术开发中心、检测中心和骨干企业的核心作用，加强优势产业关键技术的研究、开发和应用，大力培育名牌，推动特色工业创新发展。大力实施人才强市战略，加快引进创新科研人才，发挥人才引领先导作用。设立北京科技大学云浮研究院。

深化政银企合作。继续落实已签约的授信额度，做实各类融资平台，增强金融对特色产业发展和100项重点工程（项目）建设的保障能力。

三、全力加快农村改革，统筹城乡区域发展

以建设农村改革发展试验区为载体，全力加快农村改革发展。实施体制机制创新工程，进一步在主体功能区规划建设、富县强镇事权改革、农村社会保障体系建设、城乡公共服务均等化、农村金融发展和社会管理、城乡土地流转、户籍管理制度改革等方面加大力度，以体制机制的新突破，进一步激发农村发展活力，加快推进城乡统筹发展。

建设农业强市。实施现代农业提升工程。积极打造“国家级畜牧业产业化示范中心”，用好温氏品牌，做大做强畜牧业，带动农民增收致富，年内培育发展省级以上农业龙头企业 2 家、市级农业龙头企业 10 家、农民专业合作社30个，新增“三品”认证22个。着力在发展高品质、高附加值的农产品上做活、做好生态农业文章，建设现代标准农田1万亩。完成林业重点生态工程面积 5.75万亩，森林抚育5.37万亩，深入推进集体林权制度改革。积极发展农林产品深加工产业。继续完善农情研判以及农业技术服务体系，切实提高服务“三农”的能力。确保粮食播种面积在180万亩，总产稳定在68万吨以上。

推进主体功能区规划。加快推进“三规合一”，制定完善全市和各县（市、区）主体功能区规划，推广“云安经验”，科学确定各区域主体功能定位。建立健全与主体功能区规划相适应的政绩考评、财政保障等机制，重点以镇为单位实行各有侧重、差别化的绩效考核措施。建立共建共享机制，打破行政区划配置资源，建立促进区域重大项目和产业发展机制，协调城乡统筹的利益关系，调动各地各方积极性。

统筹县域协调发展。以加快发展县域经济为重点，统筹推进各县（市、区）协调发展。大力支持各地依托资源禀赋和传统优势发展壮大特色产业。抓住全省开展交通建设大会战的机遇，加大各地交通基础设施建设投入。推进富县强镇事权改革，落实财政体制改革，加强县级政府提供基本公共服务的财力保障。加快特色县城和中心镇发展，提高城镇综合管理和服务水平，增强群众对居住地的认同感和归属感。

启动“信用云浮”建设。实施信用云浮成长工程。以建设全省信用体系试点市为载体，以农村信用体系建设为突破口，制定实施方案，在全市推广“郁南经验”，开展创建“信用云浮”活动，倡导诚实守信、互信互助的社会风气，着力打造云浮人讲信用守承诺、云浮无假货、“云浮出品”信得过的“云浮信用”品牌，助推全市经济社会跨越发展。

四、着力建设文化强市，提升文化影响力

加强社会主义核心价值体系宣传教育，大力弘扬云浮优秀文化传统，加快培育健康、生态、幸福的生活方式，加快构建覆盖城乡的公共文化服务体系，积极打造地方传统特色文化品牌，全面推进以文强市、以文兴市、以文旺市。

优先发展教育。实施教育提升计划。加快普及高中阶段教育。推进市教育园区建设，加快市技工学校实训基地、云安县凤凰高级中学等项目建设进度，云浮市实验学校年内动工。新建一所市示范性幼儿园，年内实现70% 的镇建有中心幼儿园，三年内实现每个镇都建有一所中心幼儿园。加快推进义务教育规范化学校建设。大力支持民办教育发展，加快发展特殊教育。落实进城务工人员随迁子女入读义务教育阶段学校政策，落实生均公用经费政策和各级各类学校助学政策。

大力发展文化事业和文化产业。实施文化惠民计划。构建普惠型公共文化服务体系，完善基层公共文化设施网络，提升公共文化服务水平。发展特色文化产业，加快广东禅文化创意产业园、石文化创意产业园、南江文化创意园建设，举办第二届广东（云浮）石文化节和广东石艺精品展、南江旅游文化节等活动，擦亮禅宗六祖文化、石艺文化、南江文化三大品牌。加强文物史迹保护开发，推进非物质文化遗产保护和研究。加强广播电视、新闻出版、版权和文化市场管理工作。完善或新建一批市县文化馆、图书馆、博物馆以及乡镇综合文化站、行政村（社区）文化室。

大力发展体育事业。全面贯彻落实《全民健身条例》，广泛开展全民健身运动。筹办市第四届运动会。年内建成市体育中心，完善和新建一批乡镇农民体育健身工程，鼓励和引导群众利用慢行系统、健身路径等开展休闲健身活动。

五、着力发展各项事业，切实改善民生福祉

保障和改善民生是加快转型升级、建设幸福云浮的出发点和落脚点。要大力发展社会各项事业，加快推进基本公共服务均等化，努力增强人民群众的幸福感。

着力推进扶贫开发。实施扶贫开发计划，深入推进产业、智力、金融和保障等扶贫，加快贫困人口脱贫速度。继续推进“大禹杯”山水田林路综合整治。继续开展好“扶贫济困日”活动。

稳定和扩大就业。实施收入倍增计划。多渠道开发就业岗位，落实和完善就业、创业政策，通过实施谋发展扩就业、推政策促就业、优服务助就业和扶持创业带动就业四项计划，力争年内新增就业岗位3万个；做细做实“八项对接”服务，实现农村劳动力有序化技能化规模化转移就业。继续加强市、县、镇（街）三级公共就业服务机构建设，让就业服务信息通过三网融合与千家万户无缝对接，保障更多人有“饭碗”。加强人文关怀，维护劳动者合法权益，营造和谐劳动关系。

健全社会保障体系。实施社保扩面计划，落实城乡最低生活保障、农村五保、社会救助、住房保障等措施，提高社会保障水平。加快推进统筹城乡居民医疗保险一体化工作，落实解决困难国有、集体企业退休人员医保问题，把所有大学生和中职技校学生全部纳入城镇居民医保。落实孤儿最低养育标准。优先发展社会养老，加快残疾人事业和慈善事业发展。

加快卫生事业发展。实施医疗健康计划。深化医药卫生体制改革，提高新型农村合作医疗保障水平，继续推进9项基本公共卫生服务项目和6项重大项目建设，全面完成229个农村卫生站规范化建设。大力推进农村改厕工作，年内完成1.4万户农村改厕任务。全市县级综合医院和中心镇卫生院实现远程医疗服务。市妇幼保健院新院年内动工建设。大力推进省级卫生镇和卫生村创建工作。加强食品药品安全、疾病防控、卫生监督和卫生应急工作。大力发展中医药事业，加快创建全国农村中医药工作先进单位。

推进平安云浮建设。实施平安云浮计划。抓好社会治安综合治理，继续深入推进社会矛盾化解、社会管理创新、公正廉洁执法“三项重点工作”，进一步做好深圳大运会安保工作，不断完善社会稳定风险评估机制和社会治安防控体系建设。坚持市领导信访接待日制度和领导包案制度，及时解决群众反映的热点难点问题，有效预防和妥善处置各类群体性事件。落实企业安全生产主体责任，实行领导干部安全生产工作“一岗双责”和“一票否决”制度，加强应急管理、救援体系建设，提高处置突发重大公共事件和自然灾害的能力。

深化重点领域关键环节改革。继续深化行政体制改革，完善公共财政和投资体制，推进国有企业和金融、价格及要素市场改革。坚持依法治税，盘活国有资产，努力增加财政收入。完善预算编制和执行管理与监督制度，坚持科学理财，切实减少不必要的行政开支，新增财力向民生事业倾斜。积极推进事业单位分类和公务用车改革。推进农村信用社定向合作和改革。加强金融风险防范和处置。健全土地、资本、人力资源、技术等要素市场。稳妥推进价格改革。

抓好人口计生、节能减排、节约集约用地、环保等工作。突出抓好农村和流动人口计生工作重点，推进综合改革，稳定低生育水平，全面完成人口和计划生育目标管理任务。大力发展循环经济，着力推进资源节约型、环境友好型社会建设。强化节能降耗，落实节能目标责任制和考核评价制度，深入实施重点节能工程。强化污染减排目标责任制，严格执行主要污染物排放总量控制和前置审核制度。加强饮用水源水质保护，推进农村和城乡结合部环境整治。加强生态建设，扎实推进节约集约用地。落实最严格的耕地保护和节约用地制度，积极推进开发补充耕地工作，加强地质勘查工作。实施社会管理创新计划，推进社会创新体制改革，健全基层管理和服务体系，规范和发展社会组织，完善基层民主管理制度。加强人民防空建设，深化创建双拥模范城工作，抓好民兵预备役建设和拥军优属、优抚安置工作。继续加强统计、人事人才、知识产权、编制、民族宗教、档案、外事侨务、港澳事务、台务、地方志、消防、防震减灾、保密、气象、工会、青年、妇儿、侨联、文联、残联、社科联、工商联、科协、红十字会、贸促会以及驻市外机构等工作。

各位代表，保障和改善民生，解决关系人民群众切身利益的热点和难点问题，是建设“幸福云浮”的重头戏。在这里，我代表市人民政府承诺，今年努力为人民群众办好十件实事：一是实现新型农村养老保险县（市、区）全覆盖。二是实现贫困人口年内脱贫率达70%以上。三是加大促进就业力度。年内新增就业岗位3万个，组织技能培训3.7万人，新增转移就业6万人，转移就业率不低于85%。四是加快推进基本公共教育服务均等化。高中阶段毛入学率达到85%以上，完成教育园区一期工程，推进远程教育发展。五是建成市人民医院新院，推进远程医疗发展，在全市所有镇卫生院和社区卫生服务机构实施国家基本药物制度。六是加大农村饮水、道路建设投入。建设400公里通500人以上自然村以及敬老院、学校、农业示范基地的农村公路。新增解决农村6.9万人的饮水安全。七是完成市中心城区供水管网改造工程。八是慢行系统向市区延伸，逐步打通中心城区断头路，完善南山森林公园和中心城区公共场所的配套设施。九是改善困难群众居住条件。建设保障性住房787套，加快推进农村贫困户危房、泥砖房改造。十是建设100间村（社区）文化室、10个以上镇综合文化站，在农村放电影1万场，农家书屋扩展到730家以上，送戏下乡100场，全市文化馆（站）、博物馆、图书馆、美术馆实行全免费开放。

六、着力加快职能转变，提高政府行政效能

加强民主法制建设，加快政府职能转变是建设幸福云浮的重要保障。要优化政府运行机制，大力推进服务型政府建设，提高各级各部门推动科学发展的能力和水平。

建设法治政府。实施基层善治计划。进一步健全“党委领导、政府负责、社会协同、公众参与”的社会管理格局，完善城乡社区和基层民主政治建设，努力提高依法治市水平。坚持依法行政，规范行政执法程序，落实行政问责制。坚持和完善向人大及其常委会报告工作、向人民政协通报情况制度，自觉接受监督。认真办理人大代表议案、建议和政协提案。启动“六五”普法。

建设服务政府。推行简政放权。继续精简行政审批事项，减少审批环节，提高审批效率。创新服务方式，进一步完善电子政务、电子监察、网上审批和“一条龙、一站式”服务，提高办事效率。实施创业人才集聚工程，统筹推进党政人才、经营管理人才、专业技术人才、社会工作人才等各类人才队伍建设。积极参与“书香大西关”读书讲坛活动，全力创建学习型机关。大力倡导深入基层调查研究和抓落实的良好政风。

建设廉洁政府。推进反腐倡廉制度建设，规范权力运行。加强审计监督和行政监察，强化经济责任审计和绩效审计。加强公车、接待、出境出国等方面的管理，节约公务开支。

各位代表！加快发展时不我待，只争朝夕。让我们在市委的坚强领导下，锐意创新，开拓进取，全面完成今年的目标任务，为“十二五”规划的实施开好局、起好步，为推动全市经济社会科学发展、跨越发展、和谐发展，建设广东富庶文明大西关和幸福云浮作出新的更大的贡献，以优异成绩迎接建党90周年！
